# Allergens induce enhanced bronchoconstriction and leukotriene production in C5 deficient mice

**DOI:** 10.1186/1465-9921-7-129

**Published:** 2006-10-17

**Authors:** Laura McKinley, Jiyoun Kim, Gerald L Bolgos, Javed Siddiqui, Daniel G Remick

**Affiliations:** 1 Department of Pathology, University of Michigan, Ann Arbor, MI, USA; 2 Boston University School of Medicine, Department of Pathology, 670 Albany Street, Room 407, Boston, MA 02118, USA

## Abstract

**Background:**

Previous genetic analysis has shown that a deletion in the complement component 5 gene-coding region renders mice more susceptible to allergen-induced airway hyperresponsiveness (AHR) due to reduced IL-12 production. We investigated the role of complement in a murine model of asthma-like pulmonary inflammation.

**Methods:**

In order to evaluate the role of complement B10 mice either sufficient or deficient in C5 were studied. Both groups of mice immunized and challenged with a house dust extract (HDE) containing high levels of cockroach allergens. Airways hyper-reactivity was determined with whole-body plesthysmography. Bronchoalveolar lavage (BAL) was performed to determine pulmonary cellular recruitment and measure inflammatory mediators. Lung homogenates were assayed for mediators and plasma levels of IgE determined. Pulmonary histology was also evaluated.

**Results:**

C5-deficient mice showed enhanced AHR to methylcholine challenge, 474% and 91% increase above baseline Penh in C5-deficient and C5-sufficient mice respectively, p < 0.001. IL-12 levels in the lung homogenate (LH) were only slightly reduced and BAL IL-12 was comparable in C5-sufficient and C5-deficient mice. However, C5-deficient mice had significantly higher cysteinyl-leukotriene levels in the BAL fluid, 1913 +/- 246 pg/ml in C5d and 756 +/- 232 pg/ml in C5-sufficient, p = 0.003.

**Conclusion:**

These data demonstrate that C5-deficient mice show enhanced AHR due to increased production of cysteinyl-leukotrienes.

## Background

The incidence and severity of allergic asthma has been steadily rising over the past 20 years. Allergic asthma results from an inappropriate immune response to inhaled allergens characterized by the recruitment of eosinophils, lymphocytes, and neutrophils to the lung, inflammatory mediator release, IgE production, mucin hypersecretion and bronchoconstriction. It is believed that CD4^+ ^Th2-type cells mediate these responses through the production of the cytokines IL-4, IL-5, IL-9 and IL-13 (Reviewed in [1]).

Recent studies have shown that patients with asthma have increased activation of complement components C3 and C5 with increased bronchalveolar lavage C3a and C5a levels following allergen challenge [2] and patients with severe asthma have higher C3a plasma levels than those with stable asthma. Numerous studies investigating the role of C3 in asthma have shown C3-deficient mice have consistently decreased AHR [3-6]. Studies by Drouin et al found C3-deficent mice have decreased airway eosinophilia, allergen-specific IgE and a reduced number of IL-4 producing cells [6]. Converse to these studies genetic analysis has inversely linked gene expression of complement component 5 (C5) with susceptibility to AHR in mice [7]. In these studies mouse strains that are more susceptible to allergen-induced AHR had decreased C5 gene expression while those resistant to developing AHR had increased C5 gene expression. Few studies have further investigated this paradigm.

In the present studies, C5-sufficient and C5-deficient congenic mice were immunized and challenged with a HDE containing high levels of cockroach allergens (CRA). CRA has been implicated as a key allergen in the incidence and severity of allergic asthma [8]. We show that C5-deficient mice have significantly increased AHR. C5-deficient mice also have a dramatic increase in BAL fluid cysteinyl-leukotriene production providing a potential mechanism for the increased AHR seen in these mice.

## Materials and methods

### Animals

6–8 week old C5-deficient (B10.D2-H2d H2T18c Hc^0^/oSnJ) and C5-sufficient (B10.D2-H2d H2T18c Hc^1^/nSnJ) congenic mice were purchased from Jackson Labs (Bar Harbor, Maine). C5-deficient mice carry a null hemolytic component (Hc) allele rendering them unable to produce C5 [9,10]. Animals were kept under standard laboratory conditions in a temperature-controlled room with a 12-hour light/dark cycle and allowed food and water ad libitum. All experiments have been approved by the University of Michigan Animal Use Committee.

### House dust collection and extraction

Dust was collected from the kitchen area of homes of asthmatic children using a vacuum with a dust collector (Indoor Biotech, Charlottesville, VA). Sterile PBS was added to the dust and mixed overnight at 4° on a rotator. Samples were centrifuged for 10 minutes at 1000 × g, 4°C. The supernatant was collected and centrifuged for 10 minutes at 1000 × g, 4°C and the HDE was stored in aliquots at -70°C for analysis of allergen content[11] The levels of cockroach allergens Bla g1 and Bla g2 in the HDE were determined using matched-antibody pairs (Indoor Biotechnologies, Charlottesville, VA). HDE contains 1,052-ng/ml Bla g1 and 1,571-ng/ml Bla g2. HDE was diluted 1:10 and heat-inactivated for 30 minutes at 57°C prior to use.

### Induction of asthma

HDE diluted 1:10 in sterile PBS was emulsified in TiterMax Gold adjuvant (1:1, CytRx, Norcross, GA). 100 μl of HDE:adjuvant was injected i.p. on day 0. On days 14 and 21 animals were challenged intratracheally with 1:10 diluted HDE, as previously described [11]. Briefly, mice were anesthetized with isoflurane (Baxter, Deerfield, IL) and the anesthetized mouse was suspended from its front incisors on an inclined board with the base of the tail taped to support its weight. The tongue was retracted using forceps while holding the jaw open and two, 25 μl aliquots of 1:10 diluted HDE were pipetted at the base of the oropharynx. Mice were sacrificed by cervical dislocation on day 22 or 23, as indicated in the text. Following exsanguination, lungs were lavaged with 2, 1-ml fractions of HBSS. The two washes were centrifuged at 1000-× g for 5 minutes. The supernatant of the first wash was saved for later analysis while the supernatant of the 2^nd ^fraction was discarded. The cell pellets from both washes were combined and total cells were counted using a Coulter counter model ZF (Coulter Electronics, Hialeah, FL). Slides of BAL fluid cells were prepared in a cytospin (Shandon Southern Instruments, Sewickley, PA), slides were stained with Diff-Quick (Baxter, Detroit, MI) and differentials were calculated by counting 300 cells. For lung homogenate preparations, the whole right lung was removed and kept on ice in 3-ml of Hanks balanced salt solution without magnesium or calcium containing one Complete protease inhibitor tablet (Roche, Mannheim, Germany) and 0.01% Triton-X. Samples were homogenized, sonicated and centrifuged at 16,000-× g for 15 minutes at 4°C. Supernatant was stored at -70°C for later protein determination.

### Airway hyperresponsiveness

AHR of mice was measured 24-hours after the last airway challenge using a whole-body plesthysmography system (Buxco, Troy, NY). Mice were placed in the main chamber and allowed to acclimate for 10 minutes. Baseline values were then recorded for 5 minutes. Mice were subsequently challenged with aerosolized PBS or increasing doses of aerosolized β-methylcholine (Sigma, St. Louis, MO) for a 2-minute period. The response was recorded for 5 minutes after each dose by measuring the pressure differences between the main chamber containing the mouse and a reference chamber. Differences in signal quantified during respiratory cycle are reflected by enhanced pause (Penh). Penh is widely used in models of pulmonary dysfunction and has been shown to correlate with airway resistance [12].

Penh values are normalized as the percent increase of average Penh for each methylcholine dose over the average Penh for PBS aerosolization. Calculating the change in Penh will account for pressure changes resultant from heating and humidification of air traveling between the chamber and lungs, termed gas conditioning. If gas conditioning is present, then an absolute Penh may not be indicative of bronchial constriction [13]. Our experimental design ensures that the Penh does reflect increases in bronchial constriction.

### Leukotriene receptor antagonist treatment protocol

Immunized, C5-deficient mice were by oro-gavage with 10 mg/kg body weight of Montelukast (Singular^®^, Merck, Whitehouse station, NJ) in 0.1 ml of PBS. The gavage was performed with plastic gastric feeding needle (Instech, Plymouth Meeting, PA) one hour before each pulmonary challenge on days 14 and 21. Control mice received 0.1 ml of PBS.

### Histology

Lungs were fixed in 10% neutral buffered formalin and processed in paraffin. Slides were prepared, stained with hematoxylin and eosin and evaluated in a blinded manner by a board certified pathologist (DGR).

### Cytokine, IgE and tryptase ELISAs

Lung homogenate and BAL fluid protein levels were assessed as previously described [14]. CCL11, CCL2, IL-12 and IL-13 primary and secondary antibodies and standards were purchased from (R & D systems, Minneapolis, MN). IL-12 was measured using antibodies directed against the p70 form (also from R & D Systems). Total plasma IgE was measured by ELISA using rat anti-mouse IgE MAb, rat anti-mouse IgE biotinylated antibody, and mouse IgE standard (Pharmingen, San Diego, CA). Standards and samples were diluted in dilution buffer containing 10% Casein, 0.05% Tween 20 and 0.0001% BSA in 1× PBS. HRP-conjugated streptavidin (Jackson ImmunoReseach Laboratories, West Grove, PA) was diluted 1:20,000 and added to plates for 30 minutes at room temperature. Color was developed with 3, 3'-5, 5' tetramethylbenzidine and the reaction was stopped with 1.5 M H_2_SO_4_. Plates were read at 465 and 590 nm in a microplate reader (Bio-Tek Instruments, Winooski, VT).

Mouse tryptase was measured by direct ELISA. Anti mouse Tryptase β-1 capture antibody and mouse tryptase β-1 standards were purchased from R&D Systems, Inc. (Minneapolis, MN). Ninety-six well plates (Nunc Immunoplate Maxisorb; Nunc, Neptune, NJ) were coated with serially diluted standard or samples, and incubated overnight at room temperature. The plates were then washed using a wash buffer containing 0.05% Tween 20 (FisherBiotech, Fair Lawn, NJ) in PBS. Nonspecific binding sites were blocked by incubating the plates with Blocker Casein (Pierce, Rockford, IL) in PBS for 1 hour. This and subsequent incubations were conducted at room temperature on a shaker. After washing, the capture antibody was added and the plates incubated for 2 h. Standard curves were prepared using the appropriate recombinant allergen. All standard and sample dilutions were made in dilution medium (10% casein in PBS supplemented with 0.1% bovine serum albumin and 0.05% Tween 20 (FisherBiotech)). Plates were washed and peroxidase-conjugated mouse anti-rat IgG (Jackson ImmunoResearch Laboratories, West Grove, PA) was added to each well. Plates were incubated for 1 h. After a final wash, 3,3',5,5' tetramethylbenzidine(Genzyme Diagnostics, San Carlos, CA) was added, plates wereincubated in the dark for 15 min, and the reaction was stopped with 1.5 N H_2_SO_4_. Plates were read using dual wavelengths (465 and 590 nm) on the Bio-Tek microplate reader (Bio-Tek Instruments, Winooski, VT), and allergen concentrations were estimated using the recombinant allergen standard curve. The lower limit of detection was 1.0 ng/ml.

### Cysteinyl-leukotriene enzyme immunoassay

Leukotriene levels in BAL fluid were measured by Enzyme Linked Immunoassay (Cayman Chemicals, Ann Arbor, MI) at different dilutions as recommended by the Cayman protocol. Only %B/B_0 _values in the linear range were accepted, samples out of range were re-run at higher dilutions.

### C3 ELISA

96-well plates were coated with 50 μl rat anti-mouse C3 MAb (1:50 in PBS, Cell Sciences, Canton, MA) overnight at 4°C. Plates were blocked with 150 μl of 2% bovine serum albumin (BSA). Samples were diluted either 1:10 or 1:50 in dilution buffer containing 0.05% Tween 20 (FisherBiotech, Fair Lawn, NJ) and 2% fetal bovine serum and 50 μl of sample in duplicate were added to the plate. 50 μl of rabbit anti-mouse C3 PAb (1:50 in dilution buffer, Cell Sciences, Canton, MA) was added after washing. Plates were washed and 50 μl of goat anti-rabbit IgG-HRP (1:5000 in dilution buffer, Jackson ImmunoResearch Laboratories, West Grove, PA) was added. With the exception of coating antibody, all incubations were for one hour at room temperature. Color was developed with 3,3',5,5' tetramethylbenzidine and the reaction was stopped with 1.5 N H_2_SO_4_. Plates were read at 465 and 590 nm in a microplate reader (Bio-Tek Instruments, Winooski, VT).

### Statistical analysis

Student t tests were performed using GraphPad Prism version 4.0 (GraphPad Software, San Diego, CA). Values are expressed as mean ± SEM; p < 0.05 was considered significant.

## Results

### C5-deficient mice have reduced pulmonary cell recruitment

**Figure 1:**
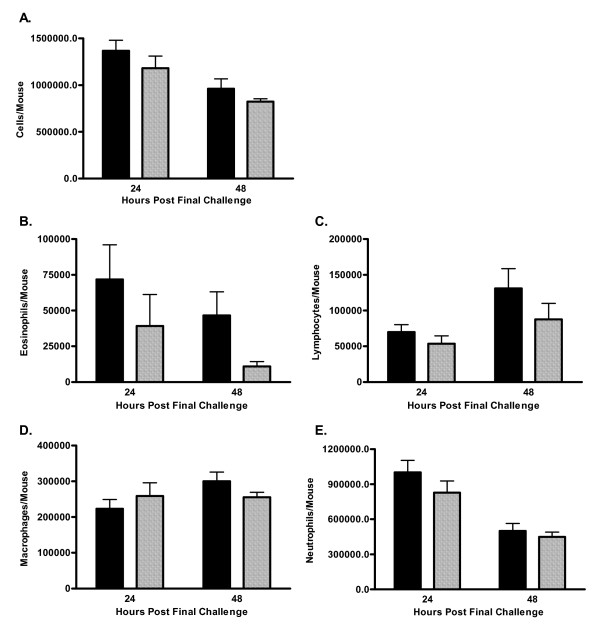
BAL fluid differential. A cell differential was performed by counting of 300 cells on prepared cytospin slides from C5-sufficient (solid bars) and C5-deficient (diagonal bars) mice. A. Total Cells; B. Eosinophils, p = 0.0618 at 48-hours; C. Lymphoctyes; D. Macrophages; E. Neutrophils. Each value is the mean ± SEM for three experiments; n = 5 mice/group at 24-hours and n = 10 mice/group at 48-hours. p > 0.05 for C5-sufficient compared to C5-deficient at each time point.

**Figure 2:**
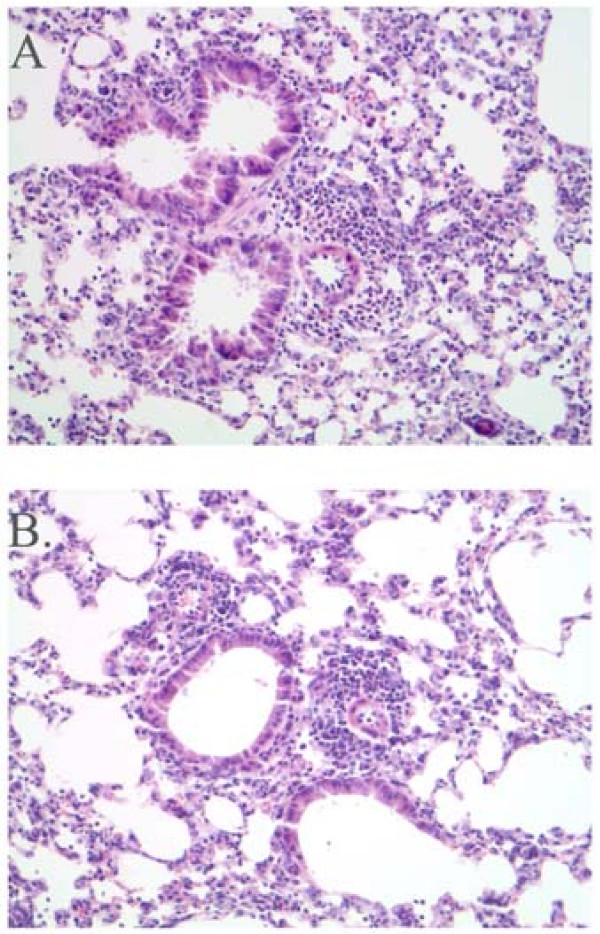
Histology. Representative H&E stained sections from C5-sufficient (A) and C5-deficient (B) animals that had been immunized and challenged with HDE and sacrificed 24-hours after the last airway challenge. Images depicted at 20×.

**Figure 3:**
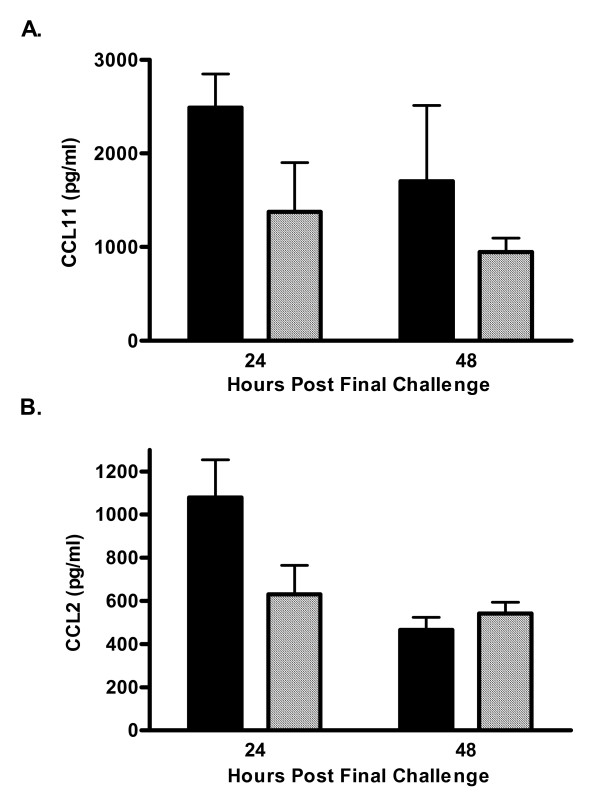
Chemokine production. Lung homogenate CCL2 and CCL11 were determined by ELISA. C5-sufficient (solid bars), C5-deficient (diagonal bars). Each value is the mean ± SEM for three experiments; n = 5 mice/group at 24-hours and n = 10 mice/group mice at 48-hours, p > 0.05 for C5-sufficient compared to C5-deficient at each time point.

House dust extracted in PBS and shown to contain high levels of cockroach allergens results in pulmonary inflammation, AHR and increased levels of lung CCL11, and plasma IgE in a mouse model of allergic asthma [11,15]. Non-immunized, i.e. naïve, C5-sufficient and C5-deficient mice had equivalent numbers of cells recovered from the BAL fluid. These cells were 99% alveolar macrophages (data not shown). C5-deficient and C5-sufficient congenic mice were immunized and challenged with HDE. AHR was assessed by whole body plethysmography on day 22, 24-hours after the last pulmonary challenge in all experiments. For the 24-hour time point animals were sacrificed immediately after AHR measurement. In separate experiments mice were sacrificed 48-hours following the last pulmonary challenge, on day 23; previous studies have shown AHR peaks at 24-hours, whereas 48-hours is the optimal time-point for eosinophil and lymphocyte influx to the airways in this model system [11,15]. Differential counting of the BAL fluid found C5-deficient mice exhibit slightly reduced total cell recruitment to the lung following allergen challenge (Figure 1A). Eosinophil influx to the airways is reduced in C5-deficient animals compared to sufficient controls, though this reduction did not reach significance (p = 0.06, Figure 1B). BAL fluid lymphocytes, macrophages, and neutrophils were comparable in the deficient and sufficient mice (Figure 1C–E). Pulmonary inflammation was also comparable between groups of animals as depicted in representative histology from an immunized and challenged C5-sufficient (Figure 2A) and deficient (Figure 2B) animal 24-hours following the last airway challenge. Blinded review of the histology slides did not indicate any significant differences between the groups. Fitting with the eosinophil influx to the airways C5-deficient mice had slightly lower levels of lung CCL11 (formerly eotaxin) in response to HDE challenge compared to sufficient controls; 2,488 ± 360 vs. 1376 ± 524 pg/ml at 24-hours (Figure 3A). Levels of the chemoattractant CCL2 (formerly MCP-1) followed a similar pattern (Figure 3B).

### Airway hyperresponsiveness is significantly increased in C5-deficient mice

**Figure 4:**
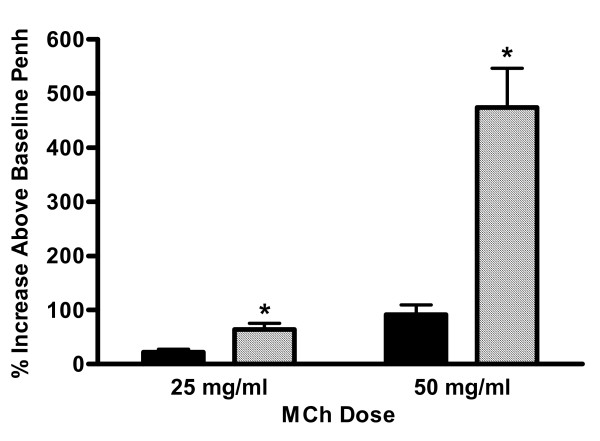
Airway hyperresponsiveness. AHR was assessed 24-hours after the last pulmonary challenge in C5-sufficient (solid bars) and C5-deficient (diagonal bars) mice. Values are reported as the percent increase in Penh of each dose of methylcholine relative to PBS aerosolization. Each value is the mean ± SEM for three experiments; n = 14 mice per group in three experiments for immunized and challenged mice. * indicates p < 0.05 for C5-sufficient compared to C5-deficient at each MCh dose, p = 0.0026 at the 25 mg/ml MCh dose and p < 0.0001 at the 50 mg/ml MCh dose.

**Figure 5:**
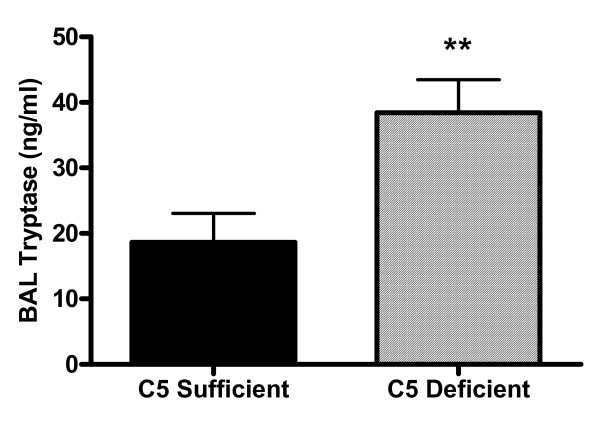
Bronchoalveolar lavage tryptase levels. Tryptase levels were determined by ELISA in the bronchoalveolar lavage fluid harvested at 48 hours after the last intratracheal challenge. C5 deficient mice had significantly higher levels of then these C5 sufficient mice. Values represent mean ± SEM with n = 11–15 for each group. **p < 0.01 compared to C5 Sufficient mice.

Non-immunized C5-sufficient and C5-deficient animals showed no difference in their response to 25 mg/ml of MCh (data not shown, 0.4 ± 2.3% increase above baseline Penh for C5-sufficient and 3.9 ± 2.8% for C5-deficient, n = 8). Mice were then immunized and challenged and AHR measured on day 22, 24-hours following the last pulmonary challenge. In accordance with the reports from the gene linkage studies, [7] C5-deficient mice had exacerbated hyperresponsiveness to a MCh challenge (Figure 4). The data is reported as a percent increase in the average Penh for each dose of nebulized MCh compared to nebulized PBS. A nebulized dose of 25 mg/ml β-Mch resulted in a 64 percent increase in Penh compared to nebulized PBS, nearly 3-fold higher than the percent increase in Penh of C5-sufficient mice (22 ± 5.9 percent increase, p = 0.0026). The exacerbated response in C5-deficient mice was even more pronounced when mice were challenged with 50 mg/ml β-Mch; 91 ± 19 vs. 474 ± 72 percent increase in C5-sufficient and C5-deficient mice respectively, p < 0.0001. Additional evidence of an increased pulmonary response in the C5 deficient mice was documented by measuring tryptase within the bronchoalveolar lavage fluid. The tryptase levels were found to be significantly elevated in the C-5 deficient animals (Figure 5).

### C5-deficient and sufficient mice produce comparable levels of BAL fluid IL-12

**Figure 6:**
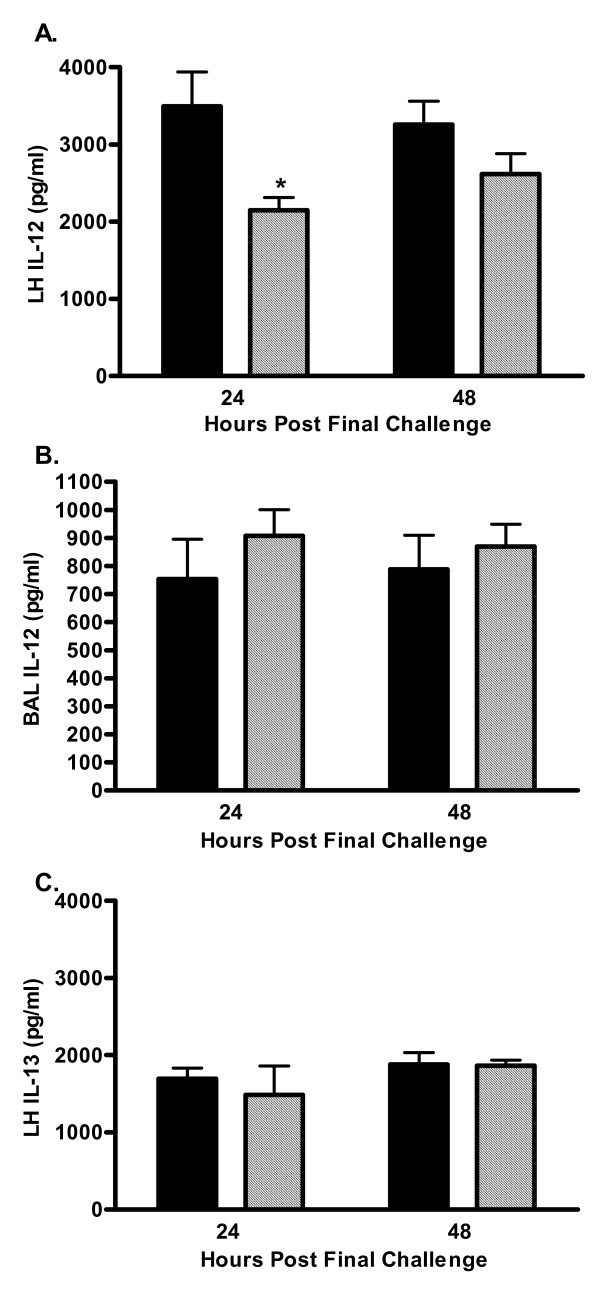
Pulmonary IL-12 p70 and IL-13. BAL fluid and lung homogenate levels of IL-12 p70 and IL-13 were measured 24- and 48-hours after the last pulmonary challenge by ELISA; C5-sufficient (solid bars) and C5-deficient (diagonal bars) mice. A. Lung homogenate IL-12 p70, * indicates p < 0.05 for C5-sufficient compared to C5-deficient at 24-hours. B. BAL fluid IL-12 p70, p > 0.05 for C5-sufficient compared to C5-deficient at each time point. C. Lung homogenate IL-13, p > 0.05 for C5-sufficient compared to C5-deficient at each time point. BAL fluid IL-13 was below detection for the assay (124 pg/ml). Each value is the mean ± SEM for three experiments; n = 5 mice/group at 24-hours and n = 10 mice/group at 48-hours.

Th2-type cytokines are believed to mediate a substantial portion of the phenotype observed in allergic asthma. It has been suggested that decreased production of the Th1-type cytokines IFN-γ and IL-12 skews the immune response to a more Th2-type response increasing susceptibility to allergic disorders (Reviewed in [1]). Previous *in vitro *studies have shown that addition of a C5a receptor antagonist to primary human monocyte cultures dose-dependently inhibited the production of the Th1 cytokine IL-12 [7]. In order to determine if C5-deficient mice had a skewed immune response due to lack of IL-12 production the levels of IL-12 were measured in the BAL fluid and lung homogenate 24 and 48 hours after the last pulmonary challenge. As shown in Figure 6A, lung homogenate IL-12 was significantly reduced in C5-deficient mice at the 24-hour time point and remained lower than sufficient animals at 48-hours. However, C5-deficient mice had BAL fluid IL-12 levels comparable to C5-sufficient mice at both 24 and 48 hours following the last pulmonary challenge (Figure 6B). IL-13 levels in the lung homogenate were also similar at both 24 and 48 hours after allergen challenge (Figure 6C); BAL fluid IL-13 was below detectable limits at these time points. These data suggest that C5-deficient animals do not have a skewed immune response favoring the allergic phenotype.

**Figure 7:**
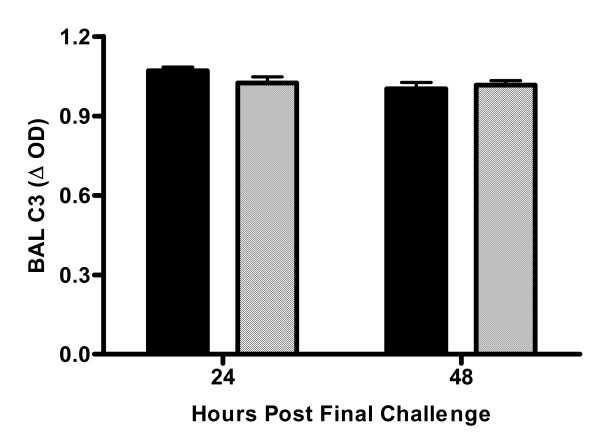
BAL fluid C3 levels. C3 levels were measured by ELISA 24 and 48-hours after the last pulmonary challenge; C5-sufficient (solid bars) and C5-deficient (diagonal bars) mice. Each value is the mean ± SEM in three experiments for n = 5 mice/group at 24-hours and n = 10 mice/group at 48-hours, p > 0.05 for C5-sufficient compared to C5-deficient at each time point.

One potential mechanism for the enhanced AHR in deficient animals is a compensatory increase in C3 activation resulting in mediator release from mast cells and basophils. Enhanced C3 activation would result in decreased levels of C3. However, C3 levels in the BAL fluid were comparable in C5-sufficient and deficient animals at 24- and 48-hours post-allergen challenge (Figure 7); due to limited sample plasma levels of C3 were not determined.

### Enhanced cysteinyl-leukotriene levels in the BAL fluid of C5-deficient mice

**Figure 8:**
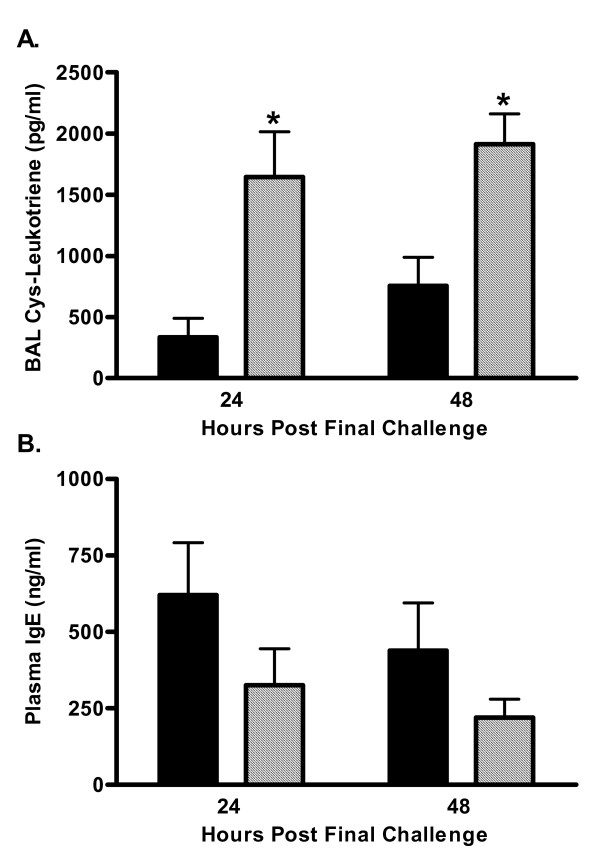
Cysteinyl-leukotriene and IgE production. A. Cysteinyl-leukotrienes were measured by immunoassay 24 and 48-hours after the last pulmonary challenge; C5-sufficient (solid bars) and C5-deficient (diagonal bars) mice. * indicates p < 0.05 for C5-sufficient compared to C5-deficient at the indicated time point, p = 0.015 at 24-hours and p = 0.003 at 48-hours. B. Total plasma IgE was determined by ELISA. Each value is the mean ± SEM for three experiments; n = 5 mice/group at 24-hours and n = 10 mice/group at 48-hours, p > 0.05 for C5-sufficient compared to C5-deficient at each time point.

Cysteinyl-leukotrienes have been implicated as a key mediator in the phenotype associated with allergic asthma including increased bronchoconstriction and mucin production [16,17]. Both C5a and C3a have receptors on mast cells and basophils and can activate these cells to release histamine and leukotrienes [18-21]. Therefore, it would be expected that animals deficient in C5 exhibit decreased leukotriene secretion and AHR. Since these animals have exacerbated airway responses to HDE we sought to determine the levels of cysteinyl-leukotrienes in the BAL fluid of deficient and sufficient mice following allergen provocation. Leukotriene levels measured at both 24- and 48-hours after the last pulmonary challenge were found to be significantly increased in C5-deficient mice (Figure 8A). C5-deficient mice had a 4-fold increase in cysteinyl-leukotiene secretion at 24-hours and this exacerbated response was still seen at 48-hours with a more than two-fold increase in BAL fluid leukotrienes in deficient animals (756 ± 232 vs. 1913 ± 246 pg/ml in C5-sufficient and C5-deficient mice respectively, p = 0.003). This enhanced leukotriene production was not the result of increased levels of IgE. C5-deficient mice had slightly lower plasma IgE levels than C5-sufficient mice (Figure 8B). Cockroach allergen specific IgE could not be measured due to lack of specific reagents.

### Leukotriene receptor antagonist prevents the augmented AHR in C5-deficient mice

**Figure 9:**
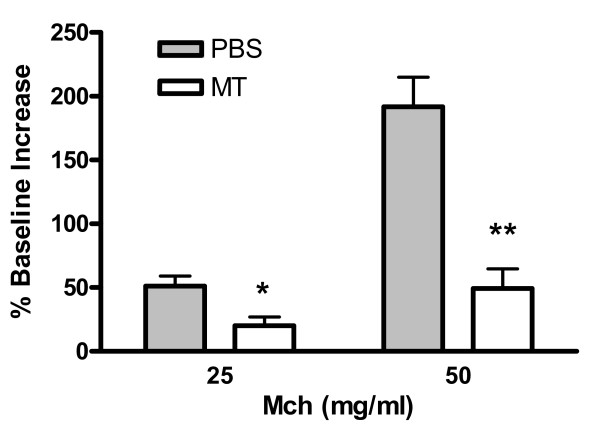
Montelukast significantly reduces airway hyperresponsiveness. Penh values were obtained from mice 24 hours after the last challenge in response to nebulized MCh via whole body plethysmography. The data are expressed as the mean ± SEM of Penh values (n = 3–4) as the increased percentage of baseline observed after PBS nebulization. * p < 0.05 and ** p < 0.01 when compared to PBS-treated group.

To prove a cause and effect relationship between the elevated pulmonary leukotriene concentrations and the enhanced AHR in the C5-deficient mice, animals were treated with the specific leukotriene receptor antagonist montelukast 1 hour prior to pulmonary challenge with the house dust extract. Blockade of the leukotriene receptor resulted in a substantial reduction in AHR. In contrast, none of the mice treated with vehicle had a decrease in AHR (Figure 9).

## Discussion

The role of the complement system in allergic asthma has recently become an area of intense investigation [22]. Complement activation in allergic asthma may occur through the classical, lectin, or alternative pathways; these pathways converge at the level of C3 activation. The anaphylatoxins C3a and C5a may also be formed by proteolytic cleavage of C3 and C5 by inflammatory cell-derived proteases or allergen proteases [22-24]. Dendritic cells, eosinophils, neutrophils, and T lymphocytes chemotax to C5a [25-28]. Furthermore, C5a mediates eosinophil and neutrophil activation resulting in degranulation and oxidant secretion [27]. In our studies, cell recruitment to the lung was not markedly decreased in C5-deficient animals suggesting the involvement of other chemotactic agents. Although these chemokines may account for allergen-induced cellular influx in these animals, levels of the chemotactic cytokines CCL11/eotaxin and CCL2/MCP-1 were not altered in C5-deficient animals. CCL11/eotaxin has been shown to be a potent eosinophil chemotactic mediator and is believed to be important in the development of allergic asthma and is a major eosinophil chemoattractant in our model [11,29,30]. Despite comparable, if not slightly reduced, pulmonary inflammation AHR was exacerbated in C5-deficient animals indicating the increased AHR in C5-deficient mice occurs independent of cellular recruitment to the airways. Previous work in our laboratory has suggested AHR is independent of inflammatory cell recruitment in that lymphocyte and eosinophil influx to the airways peaks at 48-hours after allergen challenge whereas AHR peaks at 24-hours [11]. In agreement with our studies, Corry et al have shown AHR occurs independently of eosinophil recruitment to the lung [31].

Exacerbated AHR in C5-deficient animals was first suggested in gene-linkage studies by Karp et al [7] that showed C5 gene expression inversely correlates with the magnitude of allergen-induced AHR in an ovalbumin model of allergic asthma; C5 gene expression was found to be lower in mouse strains more susceptible to the development of allergen-induced AHR. More recently, Kohl et al showed blocking C5aR signaling during initial allergen priming results in exacerbated TH2 responses and increased AHR compared to control [32]. It has been suggested that the increased AHR in C5-deficient animals is due to a skewed immune response in favor of a TH2 phenotype; mouse strains deficient in C5 (A/J) have significantly decreased IL-12 production from cultured peritoneal macrophages following IFN-γ + *Staphalococcus aureus *(SAC) stimulation compared to cultured macrophages isolated from C5-sufficient C3H/HeJ mice. Primary human monocyte cultures stimulated with IFN-γ + SAC in the presence of a specific C5aR antagonist showed a dose-dependent decrease in IL-12 production as well [7]. Taken together this suggests that C5a signaling through the C5aR would result in IL-12 production and Th1-cell differentiation; thus there is an abrogated IL-12 response in C5-deficient animals that may favor Th2-cell development and potentiate the allergic phenotype. These studies do not address the *in vivo *IL-12 response following allergen stimulation. We report C5-deficient mice immunized and challenged with HDE have significantly decreased lung homogenate IL-12 at 24-hours but similar levels of IL-12 in BAL fluid compared to C5-sufficient mice of the same genetic background. IL-13 in the lung was also comparable in sufficient and deficient animals at 24- and 48-hours after allergen challenge. These time points were chosen as optimal for the measurement of AHR and cellular influx to the airways however, the peak of cytokine and chemokine production in lung is likely a much earlier event. It is plausible that the Th1/Th2 balance is skewed in the early asthmatic response and further studies to investigate the time course of IL-12 and IL-13 production in the lung need to be performed to determine if cytokine levels are comparable early after allergen provocation.

Increased production of cysteinyl-leukotrienes relates to increased AHR [33,34] and provides a potential mechanism for the substantial increase in AHR seen in C5-deficient mice following allergen provocation. Cysteinyl-leukotrienes are secreted by activated mast cells, basophils and eosinophils [35,36]. Studies using W/W^v ^mice deficient in mast cells have shown the importance of these cells in allergen-induced AHR. Mast cell deficient animals immunized and challenged with OVA fail to develop AHR to MCh challenge but AHR is restored when mast cells are reconstituted [37,38]. Clinically, basophils and mast cells accumulate in the airways of asthmatic patients [39]. We show for the first time that C5-deficient animals have significantly increased production of cysteinyl-leukotrienes in the BAL fluid compared to sufficient controls. Furthermore, we found this enhanced cysteinyl-leukotriene production in C5-deficient animals was directly linked to the substantial increase in AHR seen in these animals. Treatment with the leukotriene inhibitor Montelukast abrogated the hyperresponsiveness. These data are interesting considering previous in vitro studies have found C5a leads to leukotriene C4 production by basophils [35], and both C3a and C5a have been shown to stimulate chemotaxis of mast cells [20]. Therefore one would expect leukotriene production to be decreased in the C5-deficient mice. The apparent dichotomy of C5aR signaling has been shown to be dependent on the time of receptor targeting. C5aR signaling at the time of sensitization has been reported to protect against the development of allergic inflammation whereas signaling through C5aR in a pre-existing inflammatory environment results in enhanced inflammatory responses. This supports our findings that the absence of C5a at the time of sensitization results in exacerbated allergic inflammatory responses [32].

The mechanism of cysteinyl-leukotriene production in C5-deficient mice is not clear from these studies and requires further investigation. Total plasma IgE is comparable in sufficient and deficient animals suggesting enhanced leukotriene production is not mediated through FcεRI. One explanation is that the degree of C3 activation is increased in animals deficient in C5. C3-deficient and C3aR-deficient mice have decreased pulmonary inflammation, Th2 cytokine production, and AHR in OVA models of allergic pulmonary inflammation and attenuated particulate matter-induced AHR [3-6,40]. These studies consistently show C3a is an important mediator of AHR. C3a binding to C3aR on mast cells and basophils could activate these cells to release inflammatory mediators including leukotrienes. We saw similar levels of C3 protein in the BAL fluid of sufficient and deficient animals after allergen challenge at 24- and 48-hours after allergen challenge and further investigation into the levels of C3 and C3a in C5-sufficient and C5-deficient animals is planned.

Alternatively, C5a and C3a may have differential roles in the allergic inflammatory response. As suggested by Hawlisch et al [22], C5a binding to C5aR results in IL-12 production while C3 binding C3aR favors Th2 cytokine production. This would explain contrasting results with C3-deficient animals and C5-deficient animals in models of allergic asthma. Previous investigators have also demonstrated that blockade of C5a will reduce late airways hyper-reactivity in an ovalbumin model [41], inhibition of complement activation also reduced airways hyper-reactivity in the ovalbumin model [42], and blockade of C5a receptors reduced airways responsiveness in an Aspergillus model [43]. The differences in experimental design between these previous reports and our studies and those of Karp [7] probably account for the divergent results. Karp's studies plus our own used genetically modified mice while the other papers inhibited complement only during the effector phase of asthma.

In conclusion, we have shown C5-deficient mice immunized and challenged with HDE containing high levels of cockroach allergens exhibit dramatically increased AHR. This AHR occurs independently of pulmonary cellular infiltration and does not appear to be the result of a skewed immune response as seen by comparable production of IL-12 in sufficient and deficient animals. It remains possible that C5-deficient mice have an abrogated IL-12 response and enhanced IL-13 production in the early phase of the asthmatic response resulting in the increased cysteinyl-leukotriene production; future studies where mice will be sacrificed in the early phase are thus planned. Increased levels of BAL fluid cysteinyl-leukotrienes seen in C5-deficient mice provide a logical mechanism for the increased level of AHR.

## Abbreviations

AHR, airway hyperresponsiveness; BAL, bronchoalveolar lavage; HDE, house dust extract; MCh, acetyl β-methylcholine; Penh, enhanced pause;
